# Supplementary material for: Epstein-Barr Virus Epitope–Major Histocompatibility Complex Interaction Combined with Convergent Recombination Drives Selection of Diverse T Cell Receptor α and β Repertoires
Source: mBio. 2020 Mar 17;11(2):e00250-20. doi: 10.1128/mBio.00250-20 (PMC7078470; doi:10.1128/mBio.00250-20)
Supplement: TABLE S1 [file mBio.00250-20-st001.pdf]

Supplementary Table 1: Characteristics of AIM donors in study population

Donor ID[1]	Gender	Time point	Elapsed time (months)[2]	EBV serology (VCA IgM)[3]	EBV viral load (log_{10}copies/10^6 B cells)	GLC[4] (%)	YVL[4] (%)
E1603	M	AIM	6	Pos	2.03	0.6	2.9
		CONV			1.92	0.5	0.7
E1632	F	AIM	7	Pos	4.36	1.1	2
		CONV			No data[5]	0.2	0.1
E1655	F	AIM	5	Pos	5.05	1.6	1.3
		CONV			3.30	0.2	0.1

[1] TCR deep sequencing was performed on tetramer sorted CD8 T cells of all three donors diagnosed with AIM and were performed at the time presentation and 5-8 months later.

[2] Time elapsed between AIM and CONV.

[3] EBV serology was only performed at AIM.

[4] Frequency of HLA-A2 restricted YVL and GLC tetramer+ cells within CD3+ CD8+ T cells sorted from PBMCs isolated from each respective donor.

[5] B cells were not available from this donor to perform a viral load assay.

AIM: acute infectious mononucleosis; CONV: convalescence; M: male; F: Female; IgM: immunoglobulin M; VCA: viral capsid antigen; Pos: positive.
