# Supplementary material for: Epstein-Barr Virus Epitope–Major Histocompatibility Complex Interaction Combined with Convergent Recombination Drives Selection of Diverse T Cell Receptor α and β Repertoires
Source: mBio. 2020 Mar 17;11(2):e00250-20. doi: 10.1128/mBio.00250-20 (PMC7078470; doi:10.1128/mBio.00250-20)
Supplement: FIG S1 [file mBio.00250-20-sf001.pdf]

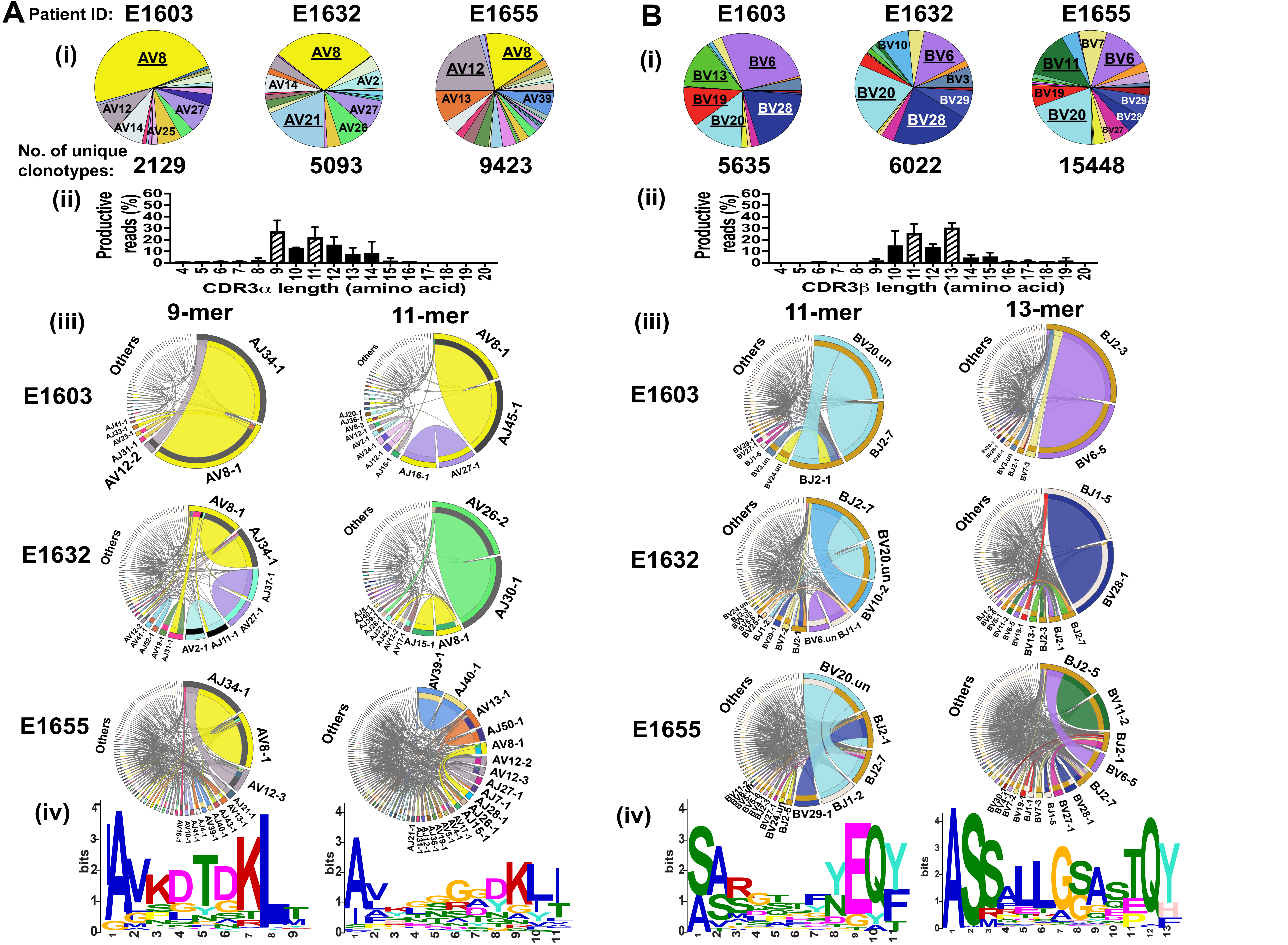

Figure S1: 9-mer TCR AV8.1-VKDTDK-AJ34 drives the selection of YVL-specific CD8 T-cells in AIM. The TCR repertoire is deconstructed by analyzing V family usage in pie chart format, CDR3 length analyses, VJ pairing by using circos plot analyses, and CDR3 amino acid motif analyses using Multiple MEME framework(77). (i) YVL-BR-specific TCRVA (A) and TRVB (B) repertoires are analyzed for 3 AIM donors (E1603, E1632, E1655) during AIM. Frequency of each TRAV (A) and TRBV (B) in total YVL-BR-specific TCR-repertoire is shown in pie charts (i). The pie plots are labeled with gene families having a frequency >5%. The total numbers of unique clonotypes in each donor is shown below the pie charts. (ii) CDR3 length distribution along with (iii) circos plots depicting V-J gene pairing and (iiii) CDR3 motif analysis for the clonotypes with the two most dominant CDR3 lengths. Genes are colored by V gene family with a fixed color sequence used throughout the manuscript.
