# Supplementary material for: Epstein-Barr Virus Epitope–Major Histocompatibility Complex Interaction Combined with Convergent Recombination Drives Selection of Diverse T Cell Receptor α and β Repertoires
Source: mBio. 2020 Mar 17;11(2):e00250-20. doi: 10.1128/mBio.00250-20 (PMC7078470; doi:10.1128/mBio.00250-20)
Supplement: FIG S2 [file mBio.00250-20-sf002.pdf]

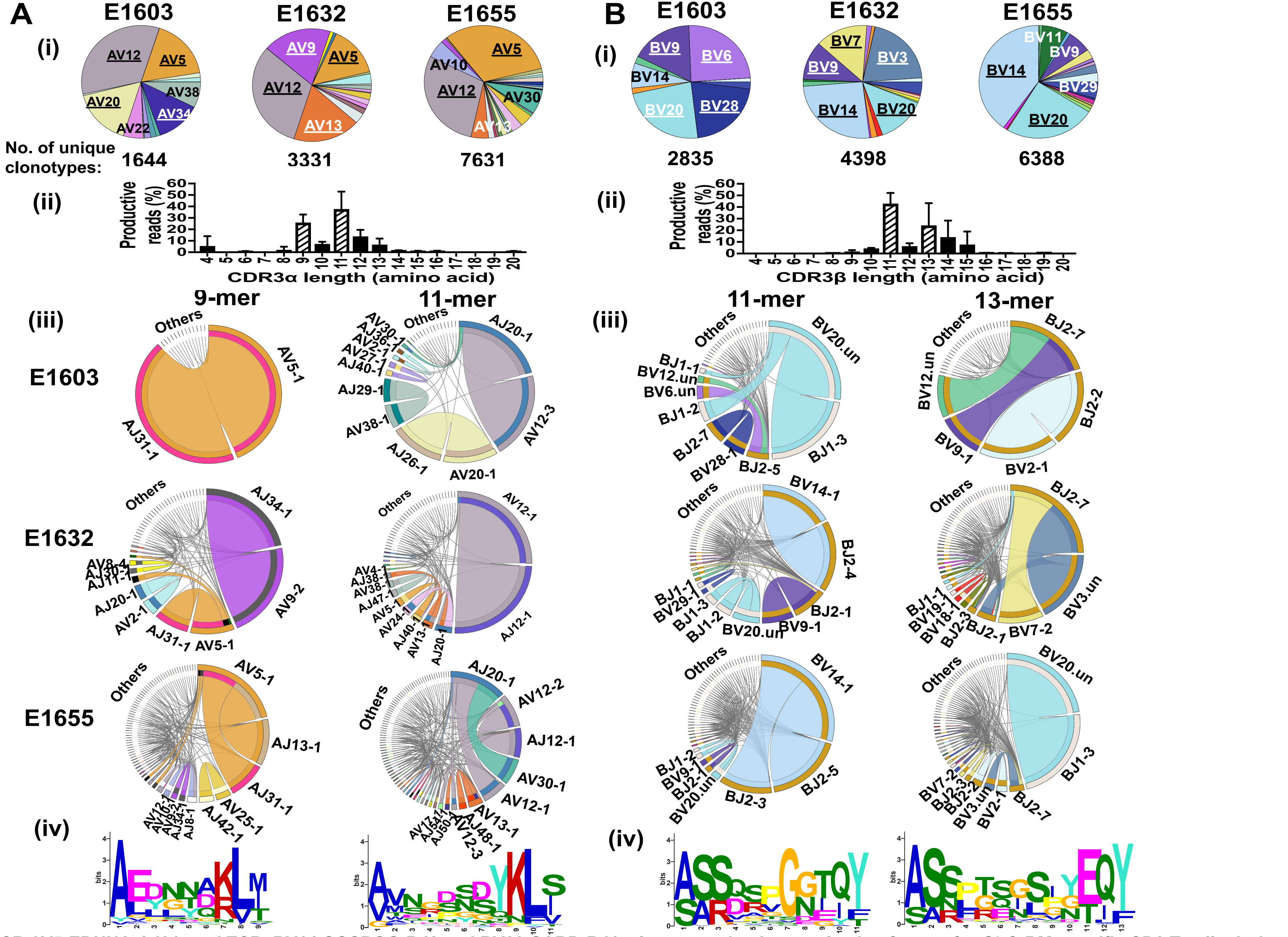

Figure S2: TCR AV5-EDNNA-AJ31, and TCR BV14-SQSPGG-BJ2 and BV20-SARD-BJ1, clones are dominant selection factors for GLC-BM-specific CD8 T-cells during AIM. GLC-BM-specific TCRVA (A) and TRVB (B) repertoires are analyzed for 3 AIM donors (E1603, E1632, E1655) during AIM. Frequency of each TRAV (A) and TRBV (B) in total GLC-BM-specific TCR-repertoire is shown in pie charts (i). The pie plots are labeled with gene families having a frequency >5%. The total numbers of unique clonotypes in each donor is shown below the pie charts. There is consistent usage of AV5 and AV12 genes in all 3 donors. There is consistent usage of BV20 in all 3 donors. Otherwise there is a high degree of variability in other AV and BV usage between donors. (ii) CDR3 length distribution along with (iii) circos plots depicting V-J gene pairing and (iiii) motif analysis for the clonotypes with the two most dominant CDR3 lengths. The frequencies of V-J combinations are displayed in circos plots, with frequency of each V or J cassette represented by its arc length and that of the V-J cassette combination by the width of the arc.
