# Supplementary material for: Epstein-Barr Virus Epitope–Major Histocompatibility Complex Interaction Combined with Convergent Recombination Drives Selection of Diverse T Cell Receptor α and β Repertoires
Source: mBio. 2020 Mar 17;11(2):e00250-20. doi: 10.1128/mBio.00250-20 (PMC7078470; doi:10.1128/mBio.00250-20)
Supplement: TABLE S2 [file mBio.00250-20-st002.pdf]

Supplementary Table S2: TCR Sequencing depth and counts of productive DNA rearrangements by donor, epitope-specificity and time point

Donor ID	Epitope-specificity	Time Point	Locus	Total reads[1]	Productive reads[2]	Fraction productive reads	Total DNA rearrangements[3]	Productive DNA rearrangements	Frequency of the most abundant DNA rearrangements
E1603	GLC	AIM	α	10,054,626	9,292,966	0.92	2,616	1,644	27.10
E1603	GLC	CONV	α	22,973,724	17,423,275	0.76	5,092	2,727	16.65
E1632	GLC	AIM	α	22,097,431	16,944,815	0.77	7,168	3,331	17.04
E1632	GLC	CONV	α	22,859,210	19,612,623	0.86	7,454	3,622	10.11
E1655	GLC	AIM	α	28,202,652	23,128,651	0.82	16,303	7,631	10.74
E1655	GLC	CONV	α	28,962,599	23,700,732	0.82	10,620	5,596	6.46
E1603	YVL	AIM	α	7,910,653	5,660,732	0.72	4,756	2,129	17.19
E1603	YVL	CONV	α	9,104,849	6,329,264	0.70	2,679	1,292	19.75
E1632	YVL	AIM	α	23,535,422	15,717,394	0.67	10,826	5,093	17.76
E1632	YVL	CONV	α	21,440,524	15,745,519	0.73	6,880	3,107	8.55
E1655	YVL	AIM	α	23,058,186	19,106,529	0.83	19,844	9,423	5.46
E1655	YVL	CONV	α	24,136,082	19,007,637	0.79	9,792	4,543	3.76
E1603	GLC	AIM	β	27,465,189	26,612,904	0.97	4,440	2,835	20.66
E1603	GLC	CONV	β	18,100,236	17,244,686	0.95	4,490	2,686	12.13
E1632	GLC	AIM	β	21,780,262	20,902,638	0.96	6,777	4,398	25.50
E1632	GLC	CONV	β	15,288,247	14,378,151	0.94	9,054	6,119	14.39
E1655	GLC	AIM	β	13,121,531	11,767,345	0.90	11,862	6,388	21.52
E1655	GLC	CONV	β	22,723,664	20,626,151	0.91	10,152	6,087	22.16
E1603	YVL	AIM	β	23,441,696	21,388,055	0.91	9,171	5,635	22.82
E1603	YVL	CONV	β	22,613,004	20,588,768	0.91	4,928	2,756	15.94
E1632	YVL	AIM	β	14,253,678	11,604,360	0.81	10,671	6,022	17.28
E1632	YVL	CONV	β	15,662,887	14,374,943	0.92	6,846	4,026	5.52
E1655	YVL	AIM	β	12,882,103	12,455,953	0.97	23,621	15,448	10.39
E1655	YVL	CONV	β	11,684,624	11,335,528	0.97	8,007	4,914	6.85

[1] Total reads are the sum of productive and non-productive reads.

[2] Productive reads are reads that do not contain premature stop codons.

[3] Total DNA rearrangements are the total number of unique reads (productive and non-productive).
