# Supplementary material for: Epstein-Barr Virus Epitope–Major Histocompatibility Complex Interaction Combined with Convergent Recombination Drives Selection of Diverse T Cell Receptor α and β Repertoires
Source: mBio. 2020 Mar 17;11(2):e00250-20. doi: 10.1128/mBio.00250-20 (PMC7078470; doi:10.1128/mBio.00250-20)
Supplement: TABLE S3 [file mBio.00250-20-st003.pdf]

Supplemental Table S3: YVL-BR specific and GLC-BM specific TRAV and TRBV dominant motifs.

A. YVL-BR specific TRAV

**T cell receptor:**	**Valpha**											
**Motif sequence:**	**"VKDTDK" (9-mer)**											
**Sample name:**	**E1603**				**E1632**				**E1655**			
**Phase of infection:**	**AIM**		**CONV**		**AIM**		**CONV**		**AIM**		**CONV**	
**Frequency of all copies (reads):**	**24,48%**(1,385,735 / 5,660,732)		**22.5 %** (1,426,914 / 6,329,264)		**10.9%** (1,718,846 / 15,717,394)		**9.7%** (1,530,475 / 15,745,519)		**5.86%** (120,799 / 19,106,529)		**0.43%** (81,323 / 19,007,637)	
**Frequency of all unique clonotypes:**	**4.1%** (195/4,756)		**6.8%** (182/2,679)		**2.67%** (290 /10,826)		**2.74%** (189/6,880)		**1.1%** (221/19844)		**0.56%** (55/9792)	
**Family usage (%):**	TRAV	TRAJ	TRAV	TRAJ	TRAV	TRAJ	TRAV	TRAJ	TRAV	TRAJ	TRAV	TRAJ
(% of usage of all other families detected for this sample)	**AV8 (85.13%)**	**AJ34 (94.87%)**	**AV8 (98.9%)**	**AJ34 (99.45%)**	**AV8 (85.52%)**	**AJ34 (91.04%)**	**AV8 (96.29%)**	**AJ34 (91%)**	**AV8 (90.5%)**	**AJ34 (90.95%)**	**AV8 (65.45%)**	**AJ34 (87.27%)**
	AV12 (11.28%)	AJ30 (2.06%)	AV35 (0.55%)	AJ45 (0.55%)	AV12 (8.97%)	AJ32 (1.72%)	AV39 (1.06%)	AJ30 (3.17%)	AV12 (3.62%)	AJ10 (1.8%)	AV12 (23.64%)	AJ50 (7.26%)
	AV3 (1.54%)	AJ32 (1.54%)	AV12 (0.55%)		AV21: 1.724%	AJ46 (1.03%)	AV2 (1.06%)	AJ46 (1.59%)	AV21 (1.36%)	AJ32 (0.9%)	AV21 (5.45%)	AJ32 (1.82%)
	AV6 (1.03%)	AJ45 (0.51%)			AV14 (1.38%)	AJ37 (1.03%)	AV25 (0.53%)	AJ32 (1.06%)	AV39 (0.9%)	AJ30 (0.9%)	AV19 (1.82%)	AJ26 (1.82%)
	AV25 (0.51%)	AJ39 (0.51%)			AV3 (1.03%)	AJ31 (1.03%)	AV14 (0.53%)	AJ59 (0.53%)	AV5 (0.9%)	AJ26 (0.9%)	AV17 (1.82%)	AJ3 (1.82%)
	AV21 (0.51%)	AJ20 (0.51%)			AV25 (0.34%)	AJ39 (0.69%)	AV3 (0.53%)	AJ45 (0.53%)	AV3 (0.9%)	AJ20 (0.9%)	AV6 (1.82%)	
					AV23 (0.34%)	AJ30 (0.69%)		AJ38 (0.53%)	AV29 (0.45%)	AJ50 (0.9%)		
					AV16 (0.34%)	AJ20 (0.69%)		AJ37 (0.53%)	AV23 (0.45%)	AJ59 (0.45%)		
					AV6 (0.34%)	AJ3 (0.69%)		AJ20 (0.53%)	AV19 (0.45%)	AJ46 (0.45%)		
						AJ59 (0.34%)		AJ3 (0.53%)	AV13 (0.45%)	AJ37 (0.45%)		
						AJ44 (0.34%)				AJ36 (0.45%)		
						AJ27 (0.34%)				AJ23 (0.45%)		
						AJ14 (0.34%)				AJ3 (0.45%)		

**Motif sequence:**	**"VREGGNFNK" (11-mer)**											
**Sample name:**	**E1603**				**E1632**				**E1655**			
**Phase of infection:**	**AIM**		**CONV**		**AIM**		**CONV**		**AIM**		**CONV**	
**Frequency of all copies (reads):**	**2.6%** (147,112 / 5,660,732)		**0.002%** (109/ 6,329,264)		**0.022%** (344 / 15,717,394)		**0.00007%** (11/ 15,745,519)		**0.0098% (1865 /19,106,529)**		**0%** (0/19,007,637)	
**Frequency of all unique clonotypes:**	**0.63%** (30/4,756)		**0.03%** (1/2,679)		**0.14%** (15/10,826)		**0.04%** (3/6,880)		**0.025%** (5/19,844)		**0%** (0/9,792)	
**Family usage (%):**	TRAV	TRAJ	TRAV	TRAJ	TRAV	TRAJ	TRAV	TRAJ	TRAV	TRAJ	TRAV	TRAJ
**(% of usage of all other families detected for this sample)**	**AV3 (76.67%)**	**AJ21 (96.66%)**	**AV3 (100%)**	**AJ21 (100%)**	**AV3 (86.67%)**	**AJ21 (86.67%)**	**AV14 (50%)**	**AJ21 (100%)**	**AV3 (60%)**	**AJ21 (100%)**	not found	not found
	AV8 (13.33%)	AJ18 (3.33%)			AV14 (6.67%)	AJ26 (13.33%)	AV3 (50%)		AV14 (20%)			
	AV14 (10%)				AV8 (6.67%)				AV8 (20%)			

B. YVL-BR specific TRBV

**T cell receptor:**	**V beta**											
**Motif sequence:**	**"SSGG" (11-mer)**											
**Sample name:**	**E1603**				**E1632**				**E1655**			
**Phase of infection:**	**AIM**		**CONV**		**AIM**		**CONV**		**AIM**		**CONV**	
**Frequency of all copies (reads):**	**0.000019%** (4 /21,388,055)		0% (0/20,588,768)		**0.023%** (2,639/11,604,360)		**0.00004%** (6/14,374,943)		**0.06%** (7,403/12,455,953)		**0.0006%** (65/11,335,528)	
**Frequency of all unique clonotypes:**	**0.018%** (1/5,635)		0% (0/2,756)		**0.4%** (24/6,022)		**0.025%** (1/4,026))		**0.15%** (24/15,448)		**0.2%** (16/4,914)	
**Family usage (%):**	TRBV	TRBJ	TRBV	TRBJ	TRBV	TRBJ	TRBV	TRBJ	TRBV	TRBJ	TRBV	TRBJ
**(% of usage of all other families detected for this sample)**	**BV6 (100%)**	**BJ2.01 (100%)**	not found	not found	BV2 (29.1%)	**BJ2.03 (54.2%)**	**BV19 (100%)**	**BJ2.01 (100%)**	**BV6 (50%)**	**BJ2.07 (87.6%)**	**BV05 (93.7%)**	**JB2.07 (100%)**
					BV27 (29.1%)	BJ2.01 (12.5%)			BV9 (8.33%)	BJ2.01 (4.1%)	BV27 (6.3%)	
					BV12 (8.33%)	BJ1.02 (4.2%)			BV12 (8.33%)	BJ2.04 (4.1%)		
					BV13 (8.33%)	BJ2.02 (4.2%)			BV13 (8.33%)	BJ2.05 (4.1%)		
					BV3 (4.19%)	BJ2.05 (4.2%)			BV3 (8.33%)			
					BV6 (4.19%)	BJ2.07 (4.2%)			BV4 (3.3%)			
					BV7 (4.19%)				BV7 (3.3%)			
					BV11 (4.19%)				BV10 (3.3%)			
					BV25 (4.19%)				BV19 (3.3%)			
					BV29 (4.19%)				BV27 (3.3%)			

**Motif sequence:**	**"LLGG" (13-mer)**											
**Sample name:**	**E1603**				**E1632**				**E1655**			
**Phase of infection:**	**AIM**		**CONV**		**AIM**		**CONV**		**AIM**		**CONV**	
**Frequency of all copies (reads):**	**0.003%** (559/21,388,055)		**0.59%** (121,787 / 20,588,768)		**17.37%** (2,016,477/11,604,360)		**6.74%** (969,516/14,374,943)		**0.84%** (104,619/12,455,953)		**0%** (0/11,335,528)	
**Frequency of all unique clonotypes:**	**0.053%** (3/5,635)		**1.16%** (32 / 2,756)		**6.4%** (388/6,022)		**2.73%** (110/4,026)		**0.36%** (56/15,448)		**0%** (0/4,914)	
**Family usage (%):**	TRBV	TRBJ	TRBV	TRBJ	TRBV	TRBJ	TRBV	TRBJ	TRBV	TRBJ	TRBV	TRBJ
**(% of usage of all other families detected for this sample)**	BV28 (33.33%)	**BJ1 (66.67%)**	**BV28 (62.5%)**	**BJ1 (100%)**	**BV28 (72.4%)**	**BJ1 (80.92%)**	**BV28 (65.45%)**	**BJ1 (100%)**	**BV6 (41%)**	**BJ2 (73.2%)**	not found	not found
	BV27 (33.33%)	BJ2 (33.33%)	BV6 (21.87%)		BV3 (19.07%)	BJ2 (19.07%)	BV6 (17.2%)		BV19 (26.78%)	BJ1 (26.8%)		
	BV6 (33.33%)		BV30 (3.1%)		BV6 (2.8%)		BV27 (2.72%)		BV28 (5.35%)			
			BV27 (3.1%)		BV27 (1.8%)		BV7 (2.72%)		BV7 (3.57%)			
			BV25 (3.1%)		BV19 (1.03%)		BV4 (2.72%)		BV3 (3.57%)			
			BV3 (6.24%)		BV7 (1.03%)		BV13 (1.8%)		BV30 (1.78%)			
					BV10 (1.02)%		BV11 (1.8%)		BV27 (1.78%)			
					BV25 (0.51%)		BV26 (0.9%)		BV25 (1.78%)			
					BV26 (0.25%)		BV25 (0.9%)		BV24 (1.78%)			
							BV19 (0.9%)		BV13 (1.78%)			
							BV12 (0.9%)		BV12 (1.78%)			
							BV10 (0.9%)		BV11 (1.78%)			
							BV3 (0.9%)		BV10 (1.78%)			
									BV5 (1.78%)			
									BV4 (1.78%)			
									BV2 (1.78%)			

C. GLC-BM specific TRAV

T cell receptor:	V alpha											
**Motif sequence:**	**"EDNNA" (9-mer)**											
**Sample name:**	**E1603**				**E1632**				**E1655**			
**Phase of infection:**	**AIM**		**CONV**		**AIM**		**CONV**		**AIM**		**CONV**	
**Frequency of all copies (reads):**	**6.5%** (604,202/9,292,966)		**9%** (1,574,269/17,423,275)		**1.2%** (203,838/16,944,815)		**4%** (785,776/19,612,623)		**0.6%** (142,712/23,128,651)		**0.8%** (198,339/23,555,245)	
**Frequency of all unique clonotypes:**	**6.4%** (105/1,644)		**9.2%** (252/2,727)		**2.2%** (74/3,331)		**4.8%** (173/3,622)		**1.4%** (113/16,303)		**0.7%** (69/10,620)	
**Family usage (%):**	TRAV	TRAJ	TRAV	TRAJ	TRAV	TRAJ	TRAV	TRAJ	TRAV	TRAJ	TRAV	TRAJ
**(% of usage of all other families detected for this sample)**	**AV5 (99.05%)**	**AJ31 (98.09%)**	**AV5 (92.46%)**	**AJ31 (97.61%)**	**AV5 (62.5%)**	**AJ31 (98.61%)**	**AV5 (81.81%)**	**AJ31 (96.97%)**	**AV5 (92.23%)**	**AJ31 (98.05%)**	**AV5 (74.62%)**	**AJ31 (95.52%)**
	AV23 (0.95%)	AJ32 (1.9%)	AV23 (2.78%)	AJ31 (1.98%)	AV13 (19.44%)	AJ13 (1.38%)	AV13 (5.45%)	AJ13 (1.21%)	AV13 (3.88%)	AJ13 (1.94%)	AV13 (10.44%)	AJ13 (2.98%)
			AV14 (1.58%)	AJ34 (0.39%)	AV14 (5.55%)		AV14 (3.03%)	AJ13 (1.21%)	AV23 (1.94%)		AV23 (7.46%)	AJ13 (1.49%)
			AV13 (1.58%)		AV9 (5.55%)		AV19 (2.42%)	AJ44 (0.6%)	AV14 (0.97%)		AV19 (4.47%)	
			AV19 (0.79%)		AV6 (5.55%)		AV9 (2.42%)		AV14 (0.97%)		AV9 (1.49%)	
			AV9 (0.79%)		AV19 (1.38%)		AV12 (1.81%)				AV9 (1.49%)	
							AV6 (1.81%)					
							AV1 (1.21%)					

**Motif sequence:**	**"VNGED" (12-mer)**											
**Sample name:**	**E1603**				**E1632**				**E1655**			
**Phase of infection:**	**AIM**		**CONV**		**AIM**		**CONV**		**AIM**		**CONV**	
**Frequency of all copies (reads):**	**6.3%** (589,714/9,292,966)		**0%** (0/17,423,275)		**3.27%** (550,296/16,944,815)		**5.6%** (1,100,592 /19,612,623)		**3.94%** (911,940/23,128,651)		**1.13%** (266,636/23,555,245)	
**Frequency of all unique clonotypes:**	**1.2%** (20/1,644)		**0%** (0/2,727)		**1.41%** (47/3,331)		**2.6%** (94/3,662)		**0.77%** (126/16,303)		**0.41%** (44/10,620)	
**Family usage (%):**	TRAV	TRAJ	TRAV	TRAJ	TRAV	TRAJ	TRAV	TRAJ	TRAV	TRAJ	TRAV	TRAJ
**(% of usage of all other families detected for this sample)**	**AV12 (90%)**	**AJ12 (100%)**	not found	not found	**AV12 (72.34%)**	**AJ12 (95.74%)**	**AV12 (76.69%)**	**AJ12 (98.05%)**	**AV12 (90.47%)**	**AJ12 (100%)**	**AV12 (72.72%)**	**AJ12 (100%)**
	AV23 (5%)				AV10 (14.89%)	AJ34 (4.24%)	AV1 (8.73%)	AJ34 (1.94%)	AV10 (4.76%)		AV10 (13.63%)	
	AV10 (5%)				AV6 (12.76%)		AV6 (7.76%)		AV24 (1.58%)		AV6 (9.09%)	
							AV21 (6.79%)		AV21 (1.58%)		AV21 (4.54%)	
									AV6 (1.58%)			

**Motif sequence:**	**"MSGSN" (11-mer)**											
**Sample name:**	**E1603**				**E1632**				**E1655**			
**Phase of infection:**	**AIM**		**CONV**		**AIM**		**CONV**		**AIM**		**CONV**	
**Frequency of all copies (reads):**	**27%** (2,523,606/9,292,966)		**16.7%** (2,906,306/17,423,275)		**0%** (0/16,944,815)		**5.43%** (1,066,456/19,612,623)		**0%** (0/23,128,651)		**0%** (0/23,555,245)	
**Frequency of all unique clonotypes:**	**14.2%** (234/1,644)		**7.73%** (211/2,727)		**0%** (0/3,331)		**2.24%** (81/3,622)		**0%** (0/16,303)		**0%** (0/10,620)	
**Family usage (%):**	TRAV	TRAJ	TRAV	TRAJ	TRAV	TRAJ	TRAV	TRAJ	TRAV	TRAJ	TRAV	TRAJ
**(% of usage of all other families detected for this sample)**	**AV12 (95.3%)**	**AJ20 (99.6%)**	**AV12 (96.7%)**	**AJ20 (95.05%)**	not found	not found	**AV12 (82.6%)**	**AJ20 (96.2%)**	not found	not found	not found	not found
	AV06 (1.3%)	AJ03 (0.4%)	AV10 (1.4%)	AJ03 (0.95%)			AV14 (7.4%)	AJ34 (2.5%)				
	AV20 (0.86%)		AV05 (0.96%)	AJ04 (0.5%)			AV21 (3.7%)	AJ39 (1.3%)				
	AV23 (0.86%)		AV23 (0.48%)	AJ34 (0.5%)			AV01 (2.5%)					
	AV05 (0.43%)		AV30 (0.48%)	AJ35 (0.5%)			AV06 (2.5%)					
	AV10 (0.43%)			AJ39 (0.5%)			AV05 (1.3%)					
	AV27 (0.43%)											
	AV34 (0.43%)											

**Motif sequence:**	**"VNGSN" (11-mer)**											
**Sample name:**	**E1603**				**E1632**				**E1655**			
**Phase of infection:**	**AIM**		**CONV**		**AIM**		**CONV**		**AIM**		**CONV**	
**Frequency of all copies (reads):**	**0.0001%** (9/9,292,966)		**0%** (0/17,424,275)		**0%** (0/16,944,815)		**0.00004%** (8/19,612,623)		**0%** (0/23,128,651)		**0%** (0/23,555,245)	
**Frequency of all unique clonotypes:**	**0.04%** (1/1,644)		**0%** (0/2,727)		**0%** (0/3,331)		**0.03%** (1/3,622)		**0%** (0/16,303)		**0%** (0/10,620)	
**Family usage (%):**	TRAV	TRAJ	TRAV	TRAJ	TRAV	TRAJ	TRAV	TRAJ	TRAV	TRAJ	TRAV	TRAJ
**(% of usage of all other families detected for this sample)**	**AV12 (100%)**	**AJ2 (100%)**	not found	not found	not found	not found	**AV12 (100%)**	**AJ2 (100%)**	not found	not found	not found	not found

D. GLC-BM specific TRBV

T cell receptor:	V beta											
**Motif sequence:**	**"SQSPGG" (11-mer)**											
**Sample name:**	**E1603**				**E1632**				**E1655**			
**Phase of infection:**	**AIM**		**CONV**		**AIM**		**CONV**		**AIM**		**CONV**	
**Frequency of all copies (reads):**	**0%** (0/26,612,904)		**0%** (0/17,244,686)		**25.6%** (5,353,658/20,902,638)		**1.49%** (214,503/14,378,151)		**39.83%** (4,687,375/11,767,345)		**40.04%** (8,258,795/20,626,151)	
**Frequency of all unique clonotypes:**	**0%** (0/2,835)		0% (0/2,686)		**6.07%** (267/4,398)		**0.47%** (29/6,119)		**9.65%** (616/6,388)		**9.72%** (592/6,087)	
**Family usage (%):**	TRBV	TRBJ	TRBV	TRBJ	TRBV	TRBJ	TRBV	TRBJ	TRBV	TRBJ	TRBV	TRBJ
**(% of usage of all other families detected for this sample)**	not found	not found	not found	not found	**BV14 (87.64%)**	**BJ2.04 (97.7%)**	**BV14: 48.276%**	**BJ2.04 (90.1%)**	**BV14 (79.7%)**	**BJ2.05 (67%)**	**BV14 (75.33%)**	**BJ2.05 (68%)**
					BV7 (1.87%)	BJ2.07 (1.1%)	BV6 (13.79%)	BJ2.01 (3.3%)	BV7 (5.19%)	BJ2.03 (32%)	BV7 (6.25%)	BJ2.03 (31%)
					BV3 (1.87%)	BJ2.01 (0.4%)	BV5 (10.34%)	BJ2.05 (3.3%)	BV6 (1.95%)	BJ2.01 (0.8%)	BV6 (2.03%)	BJ2.01 (0.8%)
					BV9 (1.49%)	BJ2.03 (0.4%)	BV28 (3.45%)	BJ2.07 (3.3%)	BV9 (1.78%)	BJ2.07 (0.2%)	BV4 (2.03%)	BJ2.07 (0.2%)
					BV6 (1.12%)	BJ2.05 (0.4%)	BV27 (3.45%)		BV3 (1.78%)		BV9 (1.86%)	
					BV5 (1.12%)		BV18 (3.44%)		BV4 (1.46%)		BV11 (1.68%)	
					BV27 (0.75%)		BV12 (3.44%)		BV11 (1.29%)		BV27 (1.52%)	
					BV21 (0.75%)		BV11 (3.44%)		BV27 (0.97%)		BV12 (1.35%)	
					BV12 (0.75%)		BV9 (3.44%)		BV23 (0.97%)		BV23 (1.18%)	
					BV11 (0.75%)		BV7 (3.44%)		BV21 (0.97%)		BV21 (1.18%)	
					BV2 (0.75%)		BV3 (3.44%)		BV25 (0.81%)		BV25 (1.01%)	
					BV25 (0.37%)				BV12 (0.81%)		BV18 (0.84%)	
					BV18 (0.37%)				BV28 (0.48%)		BV5 (0.84%)	
					BV4 (0.37%)				BV2 (0.48%)		BV03 (0.84%)	
									BV13 (0.32%)		BV2 (0.84%)	
									BV5 (0.64%)		BV13 (0.51%)	
									BV16 (0.16%)		BV28 (0.17%)	
									BV10 (0.16%)		BV24 (0.17%)	
											BV10 (0.34%)	

**Motif sequence:**	**"SPTSG" (13-mer)**											
**Sample name:**	**E1603**				**E1632**				**E1655**			
**Phase of infection:**	**AIM**		**CONV**		**AIM**		**CONV**		**AIM**		**CONV**	
**Frequency of all copies (reads):**	**0.00006%** (16/26,612,904)		**9.01%** (1,554,025/17,244,686)		**20.4%** (4,265,886/20,902,638)		**22.42%** (3,223,740/14,378,151)		**1.92%** (225,991/11,767,345)		**0.2%** (41,172/20,626,151)	
**Total frequency:**	**0.18%** (5/2,835)		**4.2%** (114/2,686)		**11.18%** (492 /4,398)		**8.75%** (536/6,119)		**1.72%** (110/6,388)		**0.74%** (45/6,087)	
**Family usage (%):**	TRBV	TRBJ	TRBV	TRBJ	TRBV	TRBJ	TRBV	TRBJ	TRBV	TRBJ	TRBV	TRBJ
**(% of usage of all other families detected for this sample)**	**BV6 (100%)**	**BJ2.05 (100%)**	**BV3 (71.05%)**	**BJ2 (99.12%)**	**BV3 (72.76%)**	**BJ2.07 (72.4%)**	**BV3 (58.21%)**	**BJ2.07 (70%)**	**BV3 (45.45%)**	**BJ2.07 (99.1%)**	**BV3 (35.55%)**	**BJ2.07 (100%)**
			BV6 (7.89%)	BJ1 (0.88%)	BV9 (5.28%)	BJ2.01 (25%)	BV6 (9.14%)	BJ2.01 (26%)	BV7 (11.82%)	BJ2.05 (0.9%)	BV7 (13.33%)	
			BV7 (5.26%)		BV7 (4.47%)	BJ2.04 (1.7%)	BV7 (8.02%)	BJ2.03 (2.3%)	BV11 (7.27%)		BV11 (11.11%)	
			BV9 (4.38%)		BV6 (3.86%)	BJ2.05 (0.9%)	BV12 (4.1%)	BJ2.05 (2%)	BV9 (6.36%)		TBV12 (6.67%)	
			BV11 (3.51%)		BV12 (2.85%)		BV4 (3.54%)	BJ2.04 (0.35%)	BV6 (5.45%)		BV4 (6.67%)	
			BV28 (1.75%)		BV14 (2.03%)		BV9 (3.36%)	BJ2.02 (0.2%)	BV12 (3.64%)		BV9 (4.44%)	
			BV27 (1.75%)		BV27 (1.42%)		BV11 (2.79%)		BV21 (2.73%)		BV6 (4.44%)	
			BV14 (1.75%)		BV23 (1.22%)		BV5 (2.61%)		BV14 (2.73%)		BV2 (4.44%)	
			BV21 (0.88%)		BV21 (1.22%)		BV27 (1.86%)		BV4 (2.73%)		BV27 (2.22%)	
			BV19 (0.88%)		BV5 (1.22%)		BV28 (1.31%)		BV2 (2.73%)		BV25 (2.22%)	
			BV15 (0.88%)		BV11 (1.02%)		BV18 (1.12%)		BV28 (1.82%)		BV21 (2.22%)	
					BV2 (0.82%)		BV14 (1.12%)		BV27 (1.82%)		BV19 (2.22%)	
					BV4 (0.61%)		BV19 (0.93%)		BV25 (1.82%)		BV18 (2.22%)	
					BV25 (0.41%)		BV21 (0.75%)		BV13 (1.82%)		BV14 (2.22%)	
					BV19 (0.41%)		BV2 (0.75%)		BV15 (0.91%)			
					BV18 (0.41%)		BV10 (0.19%)		BV5 (0.91%)			
							BV23 (0.19%)					

**Motif sequence:**	**"SARD" (11-mer)**											
**Sample name:**	**E1603**				**E1632**				**E1655**			
**Phase of infection:**	**AIM**		**CONV**		**AIM**		**CONV**		**AIM**		**CONV**	
**Frequency of all copies (reads):**	**23.42%** (6,232,244/26,612,904)		**22.79%** (3,931,496/17,244,686)		**8.64%** (1,805,070/20,902,638)		**12.15%** (1,747,217 /14,378,151)		**2.01%** (236,726/11,767,345)		**4.41%** (910,160/20,626,151)	
**Total frequency:**	**15.34%** (435/2,835)		**10.31%** (277/2,686)		**4.05%** (178 /4,398)		**2.65%** (162/6,119)		**2.81%** (180 /6,388)		**1.85%** (113 /6,087)	
**Family usage (%):**	TRBV	TRBJ	TRBV	TRBJ	TRBV	TRBJ	TRBV	TRBJ	TRBV	TRBJ	TRBV	TRBJ
**(% of usage of all other families detected for this sample)**	**BV20 (98.85%)**	**BJ1 (99.31%)**	**BV20 (97.83%)**	**BJ1 (98.92%)**	**BV20 (98.31%)**	**BJ1: 97.75%**	**BV20: 99.38%**	**BJ1 (90.74%)**	**BV20 (98.89%)**	**BJ1 (99.44%)**	**BV20 (100%)**	**BJ1 (97.34%)**
	BV29 (1.15%)	BJ2 (0.69%)	BV29 (2.17%)	BJ2 (1.08%)	BV29 (1.69%)	BJ2: 2.25%	BV29: 0.62%	BJ2 (9.26%)	BV29 (1.1)%	BJ2 (0.56%)		BJ2 (2.66%)
