# Supplementary material for: Epstein-Barr Virus Epitope–Major Histocompatibility Complex Interaction Combined with Convergent Recombination Drives Selection of Diverse T Cell Receptor α and β Repertoires
Source: mBio. 2020 Mar 17;11(2):e00250-20. doi: 10.1128/mBio.00250-20 (PMC7078470; doi:10.1128/mBio.00250-20)
Supplement: FIG S6 [file mBio.00250-20-sf006.pdf]

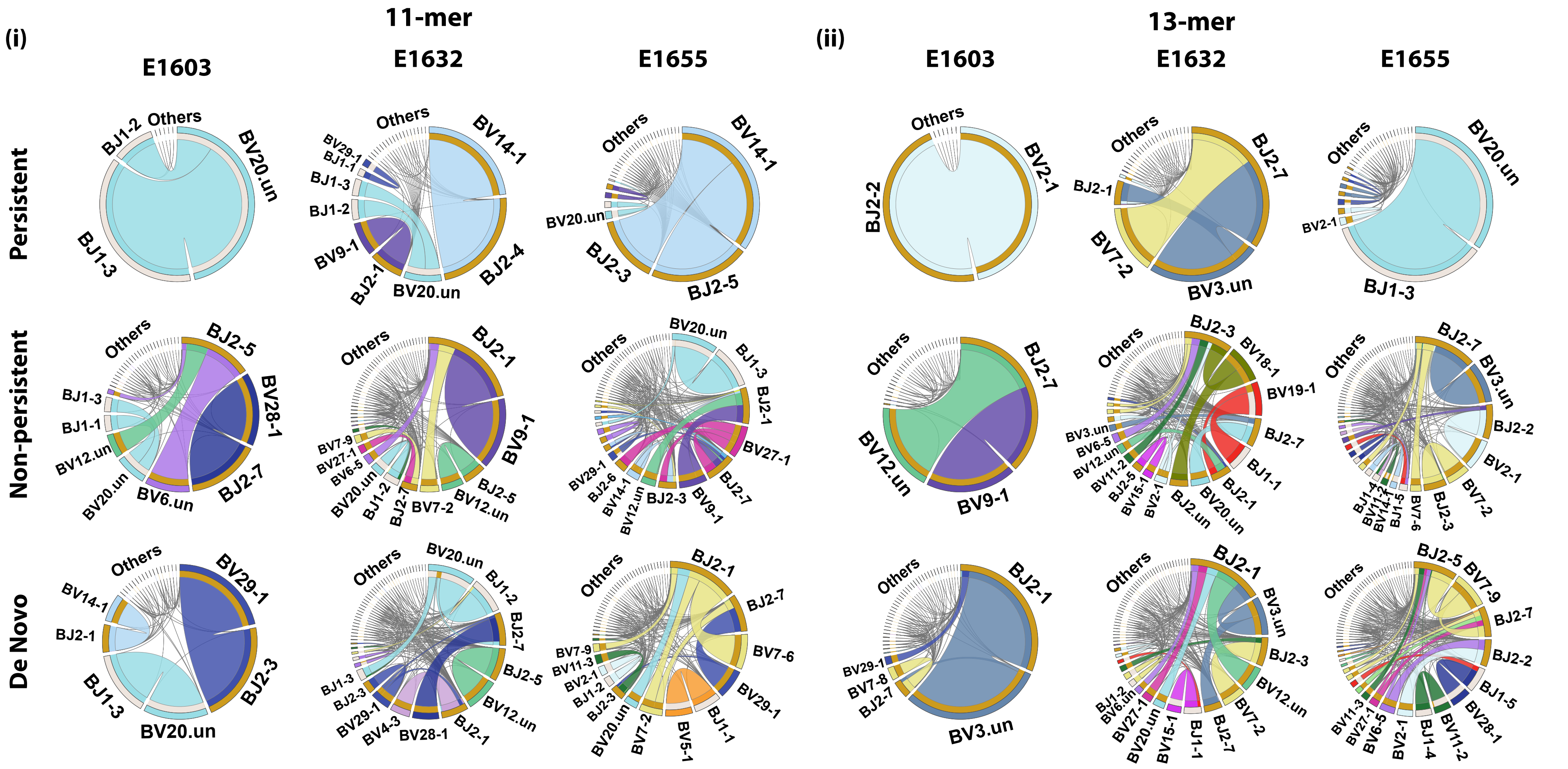


**Figure S6: Unique patterns of V-J usage for persistent, non-persistent and de novo clonotypes of 11-mer (i) and 12-mer (ii) CDR3b of the GLC-BM-specific CD8 T-cell responses. The frequencies of V-J combinations in three AIM donors for GLC-BM-specific TCRb repertoires are displayed in circos plots, with frequency of each V or J cassette represented by its arc length and that of the V-J cassette combination by the width of the arc.**
