# Supplementary material for: Epstein-Barr Virus Epitope–Major Histocompatibility Complex Interaction Combined with Convergent Recombination Drives Selection of Diverse T Cell Receptor α and β Repertoires
Source: mBio. 2020 Mar 17;11(2):e00250-20. doi: 10.1128/mBio.00250-20 (PMC7078470; doi:10.1128/mBio.00250-20)
Supplement: TABLE S4 [file mBio.00250-20-st004.pdf]

Supplemental Table S4: YVL-BR specific and GLC-BM specific TRAV and TRBV dominant motifs in persistent, non-persistent and *de novo* clonotypes.

A. YVL-BR specific TRAV (persistent clonotypes)

**T cell receptor:**	**Valpha**											
**Motif sequence:**	**Persistent "VKDTDK" (9-mer)**											
**Sample name:**	**E1603**				**E1632**				**E1655**			
**Phase of infection:**	**AIM**		**CONV**		**AIM**		**CONV**		**AIM**		**CONV**	
**Frequency of all copies (reads):**	**32.37%** (1,238,327/3,824,852)		**42.29%** (1,426,296/3,372,293)		**12.66%** (1,533,671/12,114,592)		**15.87%** (1,529,404/9,637,298)		**81.82%** (328,595/401,597)		**1.08%** (81,139/ 7,472,186)	
**Frequency of all unique clonotypes:**	**21.95%** (36/164)		**21.95%** (36/164)		**15.71%** (71/452)		**15.71%** (71/452)		**6.21%** (25/402)		**6.21%** (25/402)	
**Family usage (%):**	TRAV	TRAJ	TRAV	TRAJ	TRAV	TRAJ	TRAV	TRAJ	TRAV	TRAJ	TRAV	TRAJ
**(% of usage of all other families detected for this sample)**	**AV8-1 (97.22%)**	**AJ34-1 (94.44%)**	**AV8-1 (97.22%)**	**AJ34-1 (94.44%)**	**AV8-1 (95.77%)**	**AJ34-1 (88.73%)**	**AV8-1 (95.77%)**	**AJ34-1 (88.73%)**	**AV8-1 (80%)**	**AJ34-1 (76%)**	**AV8-1 (80%)**	**AJ34-1 (76%)**
	AV12-2 (2.78%)	AJ45-1 (5.56%)	AV12-2 (2.78%)	AJ45-1 (5.56%)	AV25 (1.4%)	AJ32-1 (2.81%)	AV25 (1.4%)	AJ32-1 (2.81%)	AV12-2 (16%)	AJ50-1 (8%)	AV12-2 (16%)	AJ50-1 (8%)
					AV14-1 (1.4%)	AJ30-1 (5.66%)	AV14-1 (1.4%)	AJ30-1 (5.66%)	AV21-1 (4%)	AJ32-1 (4%)	AV21-1 (4%)	AJ32-1 (4%)
					AV3-1 (1.4%)	AJ59 (1.4%)	AV3-1 (1.4%)	AJ59-1 (1.4%)		AJ26-1 (4%)		AJ26-1 (4%)
						AJ37 (1.4%)		AJ37-1 (1.4%)		AJ3-1 (8%)		AJ3-1 (8%)

B. YVL-BR specific TRAV (non-persistent clonotypes)

**T cell receptor:**	**Valpha**											
**Motif sequence:**	**Non-Persistent "VKDTDK" (9-mer)**											
**Sample name:**	**E1603**				**E1632**				**E1655**			
**Phase of infection:**	**AIM**		**CONV**		**AIM**		**CONV**		**AIM**		**CONV**	
**Frequency of all copies (reads):**	**8.02%** (147,408/1,835,880)		not applied		**5.14%** (185,175/3,602,802)		not applied		**5.74%** (792,204/13,797,945)		not applied	
**Frequency of all unique clonotypes:**	**8.09%** (159/1965)		not applied		**4.71%** (219/4,641)		not applied		**2.17%** (196/9,021)		not applied	
**Family usage (%):**	TRAV	TRAJ	TRAV	TRAJ	TRAV	TRAJ	TRAV	TRAJ	TRAV	TRAJ	TRAV	TRAJ
**(% of usage of all other families detected for this sample)**	**AV8-1 (82.39%)**	**AJ34-1 (94.96%)**	not applied		**AV8-1 (82.19%)**	**AJ34-1 (90.84%)**	not applied		**AV8-1 (91.84%)**	**AJ34-1 (92.86%)**	not applied	
	AV12-2 (13.20%)	AJ32 (1.88%)			AV12 (11.87%)	AJ46 (1.37%)			AV12 (2.04%)	AJ30 (1.02%)		
	AV3 (1.88%)	AJ30 (1.25%)			AV21-1 (2.28%)	AJ32 (1.37%)			AV39-1 (1.02%)	AJ20 (1.02%)		
	AV6 (1.25%)	AJ39 (0.62%)			AV14-1 (1.37%)	AJ31-1 (1.37%)			AV21 (1.02%)	AJ10 (1.02%)		
	AV25-1 (0.62%)	AJ20 (1.24%)			AV3 (0.91%)	AJ39-1 (0.91%)			AV5 (1.02%)	AJ59 (0.51%)		
	AV21-1 (0.62%)				AV23-1 (0.45%)	AJ37-1 (0.91%)			AV3 (1.02%)	AJ46 (0.51%)		
					AV16-1 (0.45%)	AJ20 (0.91%)			AV29 (0.51%)	AJ37 (0.51%)		
					AV6 (0.45%)	AJ3 (0.91%)			AV23 (0.51%)	AJ36 (0.51%)		
						AJ44 (0.45%)			AV19 (0.51%)	AJ32 (0.51%)		
						AJ27-1 (0.45%)			AV13 (0.51%)	AJ26-1 (0.51%)		
						AJ14-1 (0.45%)				AJ23 (1.2%)		

C. YVL-BR specific TRAV (*de novo* clonotypes)

**T cell receptor:**	**Valpha**											
**Motif sequence:**	**De novo "VKNTDK" (9-mer)**											
**Sample name:**	**E1603**				**E1632**				**E1655**			
**Phase of infection:**	**AIM**		**CONV**		**AIM**		**CONV**		**AIM**		**CONV**	
**Frequency of all copies (reads):**	not applied		**0.0035%** (65/1,835,880)		not applied		**0.00094%** (34/3,602,802)		not applied		**0.00025%** (34/13797945)	
**Frequency of all unique clonotypes:**	not applied		**0.4%** (8/1,965)		not applied		**0.24%** (11/4,641)		na		**0.055%** (5/9,021)	
**Family usage (%):**	TRAV	TRAJ	TRAV	TRAJ	TRAV	TRAJ	TRAV	TRAJ	TRAV	TRAJ	TRAV	TRAJ
**(% of usage of all other families detected for this sample)**	not applied		**AV8-1 (100%)**	**AJ34-1(100%)**	not applied		**AV8-1 (100%)**	**AJ34-1 (90.9%)**	not applied		**AV8-1 (100%)**	**AJ34-1(100%)**
								AJ37-1 (9.1%)				

D. YVL-BR specific TRBV (persistent clonotypes)

**T cell receptor:**	**Vbeta**											
**Motif sequence:**	**Persistent "SALLGX" (13-mer)**											
**Sample name:**	**E1603**				**E1632**				**E1655**			
**Phase of infection:**	**AIM**		**CONV**		**AIM**		**CONV**		**AIM**		**CONV**	
**Frequency of all copies (reads):**	**26.71%** (5,002,042 / 18,722,687)		**23.65%** (3,472,087/14,679,574)		not found		not found		not found		not found	
**Frequency of all unique clonotypes:**	**15.55%** (105/675)		**15.55%** (105/675)		not found		not found		not found		not found	
**Family usage (%):**	TRBV	TRBJ	TRBV	TRBJ	TRBV	TRBJ	TRBV	TRBJ	TRBV	TRBJ	TRBV	TRBJ
**(% of usage of all other families detected for this sample)**	**BV6-5 (86.66%)**	**BJ2-3 (100%)**	**BV6-5 (86.66%)**	**BJ2-3 (100%)**	not found	not found	not found	not found	not found	not found	not found	not found
	BV28-1 (1.9%)		BV28-1 (1.9%)									
	BV27-1 (1.9%)		BV27-1 (1.9%)									
	BV3un (1.9%)		BV3 (1.9%)									
	BV25-1 (0.95%)		BV25-1 (0.95%)									
	BV21-1 (0.95%)		BV21-1 (0.95%)									
	BV19-1 (0.95%)		BV19-1 (0.95%)									
	BV13-1 (0.95%)		BV13-1 (0.95%)									
	BV12 (0.95%)		BV12 (0.95%)									
	BV10-1 (0.95%)		BV10-1 (0.95%)									
	BV7-3 (0.95%)		BV7-3 (0.95%)									
	BV2 (0.95%)		BV2 (0.95%)									

E. YVL-BR specific TRBV (non-persistent clonotypes)

**T cell receptor:**	**Vbeta**											
**Motif sequence:**	**Non Persistent "SALLGX" (13-mer)**											
**Sample name:**	**E1603**				**E1632**				**E1655**			
**Phase of infection:**	**AIM**		**CONV**		**AIM**		**CONV**		**AIM**		**CONV**	
**Frequency of all copies (reads):**	**1.06%** (28,269/2,665,368)		not applied		not found		not applied		not found		not applied	
**Frequency of all unique clonotypes:**	**12.27%** (609/4,960)		not applied		not found		not applied		not found		not applied	
**Family usage (%):**	TRBV	TRBJ	TRBV	TRBJ	TRBV	TRBJ	TRBV	TRBJ	TRBV	TRBJ	TRBV	TRBJ
**(% of usage of all other families detected for this sample)**	**BV6 (94.74%)**	**BJ2 (99.84%)**	not applied		not found	not found	not applied		not found	not found	not applied	
	BV7 (2.45%)	BJ1 (0.16%)										
	BV12 (0.65%)											
	BV27-1 (0.33%)											
	BV24 (0.33%)											
	BV11 (0.33%)											
	BV5 (0.33%)											
	BV2 (0.33%)											
	BV14 (0.16%)											
	BV4 (0.16%)											
	BV3 (0.16%)											

F. YVL-BR specific TRBV (*de novo* clonotypes)

**T cell receptor:**	**Vbeta**											
**Motif sequence:**	**De Novo "SALLGX" (13-mer)**											
**Sample name:**	**E1603**				**E1632**				**E1655**			
**Phase of infection:**	**AIM**		**CONV**		**AIM**		**CONV**		**AIM**		**CONV**	
**Frequency of all copies (reads):**	not applied		**0.042%** (2,494/5,909,194)		not applied		**11.64%** (861,364/7,396,900)		not applied		not found	
**Frequency of all unique clonotypes:**	not applied		**11.72%** (244/2,081)		not applied		**4.3%** (145/3,368)		not applied		not found	
**Family usage (%):**	TRBV	TRBJ	**TRBV**	**TRBJ**	TRBV	TRBJ	TRBV	TRBJ	TRBV	TRBJ	TRBV	TRBJ
**(% of usage of all other families detected for this sample)**	not applied		**BV6 (97.95%)**	**BJ2 (99.59%)**	not applied		**BV6 (82.06%)**	**BJ2 (100%)**	not applied		not found	not found
			BV27 (0.82%)	BJ1 (0.41%)			BV10 (3.45%)					
			BV28-1 (0.41%)				BV7-6 (2.76%)					
			BV10 (0.41%)				BV28-1 (2.07%)					
			BV9 (0.41%)				BV27-1 (1.38%)					
							BV13 (1.38%)					
							BV3 (1.38%)					
							BV25 (0.69%)					
							BV24 (0.69%)					
							BV19-1 (0.69%)					
							BV12 (0.69%)					
							BV11 (0.69%)					
							BV5 (0.69%)					
							BV2 (1.38%)					

G. GLC-BM specific TRAV (persistent clonotypes)

**T cell receptor:**	**Valpha**											
**Motif sequence:**	**Persistent "EDNNA" (9-mer)**											
**Sample name:**	**E1603**				**E1632**				**E1655**			
**Phase of infection:**	**AIM**		**CONV**		**AIM**		**CONV**		**AIM**		**CONV**	
**Frequency of all copies (reads):**	**12.45%** (603,951/4,847,739)		**27.39%** (1573,133/5,742,713)		**1.55%** (181,290/1,1676,908)		**1.55%** (181,290/1,1676,908)		**0.91%** (133,331/14,630,436)		**1.77%** (198,068/11,163,342)	
**Frequency of all unique clonotypes:**	**24.57%** (29/118)		**24.57%** (29/118)		**7.87%** (26/330)		**7.87%** (26/330)		**2.6%** (21/810)		**2.6%** (21/810)	
**Family usage (%):**	TRAV	TRAJ	TRAV	TRAJ	TRAV	TRAJ	TRAV	TRAJ	TRAV	TRAJ	TRAV	TRAJ
**(% of usage of all other families detected for this sample)**	**AV5-1(96.55%)**	**AJ31-1 (100%)**	**AV5-1(96.55%)**	**AJ31-1 (100%)**	**AV5-1 (61.54%)**	**AJ31-1 (96.15%)**	**AV5-1 (61.54%)**	**AJ31-1 (96.15%)**	**AV5-1 (80.95%)**	**AJ31-1 (95.23%)**	**AV5-1 (80.95%)**	**AJ31-1 (95.23%)**
	AV23-1 (3.44%)		AV23-1 (3.44%)		AV13-1 (15.38%)	AJ13 (3.84%)	AV13-1 (15.38%)	TCRAJ13: 3.846%	AV13-1 (14.28%)	AJ13-1 (4.76%)	AV13-1 (14.28%)	AJ13-1 (4.76%)
					AV14-1 (7.69%)		AV14-1 (7.69%)		AV23-1 (4.77%)		AV23-1 (4.77%)	
					AV9-1 (7.69%)		AV9-1 (7.69%)					
					AV6 (7.69%)		AV6 (7.69%)					

H. GLC-BM specific TRAV (*de novo* clonotypes)

**T cell receptor:**	**Valpha**											
**Motif sequence:**	**De Novo "ELDGQ" (9-mer)**											
**Sample name:**	**E1603**				**E1632**				**E1655**			
**Phase of infection:**	**AIM**		**CONV**		**AIM**		**CONV**		**AIM**		**CONV**	
**Frequency of all copies (reads):**	not applied		**10.64%** (1,243,532/11,680,562)		not applied		**0.0012%** (177/14,259,876)		not applied		not found	
**Frequency of all unique clonotypes:**	not applied		**8.31%** (217/2,609)		not applied		**0.03%** (1/3,292)		not applied		not found	
**Family usage (%):**	TRAV	TRAJ	TRAV	TRAJ	TRAV	TRAJ	TRAV	TRAJ	TRAV	TRAJ	TRAV	TRAJ
**(% of usage of all other families detected for this sample)**	not applied		**AV5-1 (94%)**	**AJ16-1 (99.54%)**	not applied		**AV5-1 (100%)**	**AJ16-1 (100%)**	not applied		not found	not found
			AV23-1 (1.38%)	AJ39-1 (0.46%)								
			AV13-1 (1.38%)									
			AV19-1 (0.92%)									
			AV14-1 (0.92%)									
			AV38-1 (0.46%)									
			AV9-1 (0.92%)									

I. GLC-BM specific TRBV (persistent clonotypes)

T cell receptor:	Vbeta											
**Motif sequence:**	**Persistent "SQSPGG" (11-mer)**											
**Sample name:**	**E1603**				**E1632**				**E1655**			
**Phase of infection:**	**AIM**		**CONV**		**AIM**		**CONV**		**AIM**		**CONV**	
**Frequency of all copies (reads):**	not found		not found		**31.22%** (5,349,199/17,132,769)		**4.26%** (214,440/5,029,504)		**47.86%** (4,665,421/9,746,758)		8254039 / 15050633 = 54.841806%	
**Frequency of all unique clonotypes:**	not found		not found		**5.35%** (19/355)		**5.35%** (19/355)		**22.95%** (202/880)		202 / 880 = 22.954545%	
**Family usage (%):**	TRBV	TRBJ	TRBV	TRBJ	TRBV	TRBJ	TRBV	TRBJ	TRBV	TRBJ	TRBV	TRBJ
**(% of usage of all other families detected for this sample)**	not found	not found	not found	not found	**BV14-1 (47.36%)**	**BJ2-4 (100%)**	**BV14-1 (47.36%)**	**BJ2-4 (100%)**	**BV14-1 (57.92%)**	**BJ2-5 (100%)**	**BV14-1 (57.92%)**	**BJ2-5 (100%)**
					BV5-1 (10.52%)		BV5-1 (10.52%)		BV7.2 (8.91%)		BV7.2 (8.91%)	
					BV27 (5.26%)		BV27 (5.26%)		BV9-1 (4.45%)		BV9-1 (4.45%)	
					BV18 (5.26%)		BV18-1 (5.26%)		BV6 (4.45%)		BV6 (4.45%)	
					BV12 (5.26%)		BV12 (5.26%)		BV4-1 (3.46%)		BV4-1 (3.46%)	
					BV11 (5.26%)		BV11 (5.26%)		BV27 (2.47%)		BV27 (2.47%)	
					BV9-1 (5.26%)		BV9-1 (5.26%)		BV25 (2.47%)		BV25 (2.47%)	
					BV7-2 (5.26%)		BV7-2 (5.26%)		BV23 (2.47%)		BV23 (2.47%)	
					BV6 (5.26%)		BV6 (5.26%)		BV21 (2.47%)		BV21 (2.47%)	
					BV3-1 (5.26%)		BV3-1 (5.26%)		BV12 (2.47%)		BV12un (2.47%)	
									BV11 (2.47%)		BV11 (2.47%)	
									BV3 29.47%)		BV3 29.47%)	
									BV2 (1.48%)		BV2 (1.48%)	
									BV13 (0.99%)		BV13 (0.99%)	
									BV28 (0.49%)		BV28 (0.49%)	
									BV10 (0.49%)		BV10 (0.49%)	

J.GLC-BM specific TRBV (non-persistent clonotypes)

T cell receptor:	Vbeta											
**Motif sequence:**	**Non Persistent "SARD" (11-mer)**											
**Sample name:**	**E1603**				**E1632**				**E1655**			
**Phase of infection:**	**AIM**		**CONV**		**AIM**		**CONV**		**AIM**		**CONV**	
**Frequency of all copies (reads):**	**4.78%** (488,798 / 10,224,655)		not applied		**3.03%** (114,341 / 3,769,869)		not applied		**0.95%** (19,206 / 2,020,587)		not applied	
**Frequency of all unique clonotypes:**	**13.84%** (371 / 2,681)		not applied		**3.66%** (148 / 4,043)		not applied		**2.54%** (140 / 5,508)		not applied	
**Family usage (%):**	TRBV	TRBJ	TRBV	TRBJ	TRBV	TRBJ	TRBV	TRBJ	TRBV	TRBJ	TRBV	TRBJ
**(% of usage of all other families detected for this sample)**	**BV20 (99.46%)**	**BJ1-2 (99.73%)**	not applied		**BV20 (98.65%)**	**BJ01 (97.97%)**	not applied		**BV20 (99.28%)**	**BJ1-3 (99.28%)**	not applied	
	BV29-1 (0.54%)	BJ2 (0.27%)			BV29-1 (1.35%)	BJ2 (2.03%)			BV29-1 (0.72%)	BJ2 (0.71%)		

K. GLC-BM specific TRBV (*de novo* clonotypes)

T cell receptor:	Vbeta											
**Motif sequence:**	**De Novo "SARD" (11-mer)**											
**Sample name:**	**E1603**				**E1632**				**E1655**			
**Phase of infection:**	**AIM**		**CONV**		**AIM**		**CONV**		**AIM**		**CONV**	
**Frequency of all copies (reads):**	not applied		**4.9%** (517,874/10,580,372)		not applied		**10.14%** (948,733/9,348,647)		not applied		**0.012%** (666/ 5,575,518)	
**Frequency of all unique clonotypes:**	not applied		**8.41%** (213/2,532)		not applied		**2.3%** (132/5,764)		not applied		**1.4%** (73/5,207)	
**Family usage (%):**	TRAV	TRAJ	TRBV	TRBJ	TRAV	TRAJ	TRBV	TRBJ	TRAV	TRAJ	TRBV	TRBJ
**(% of usage of all other families detected for this sample)**	not applied		**BV20-1 (98.59%)**	**BJ1-3 (99.53%)**	not applied		**BV20-1 (98.48%)**	**BJ1 (89.39%)**	not applied		**BV20-1 (100%)**	**BJ1 (95.89%)**
			BV29-1(1.41%)	BJ2-3 (0.47%)			BV29-1 (1.52%)	BJ2-3 (10.6%)				BJ2-1 (4.11%)
